# Clinical RNA sequencing confirms compound heterozygous intronic variants in *RYR1* in a patient with congenital myopathy, respiratory failure, neonatal brain hemorrhage, and d‐transposition of the great arteries

**DOI:** 10.1002/mgg3.1804

**Published:** 2021-09-16

**Authors:** Amelle Shillington, Alonso Zea Vera, Tanya Perry, Robert Hopkin, Cameron Thomas, David Cooper, Kristen Suhrie

**Affiliations:** ^1^ Department of Human Genetics Cincinnati Children’s Hospital Medical Center Cincinnati OH USA; ^2^ Department of Neurology Cincinnati Children’s Hospital Medical Center Cincinnati OH USA; ^3^ Department of Cardiology Cincinnati Children’s Hospital Medical Center Cincinnati OH USA; ^4^ Department of Neonatology Cincinnati Children’s Hospital Medical Center Cincinnati OH USA

**Keywords:** intronic variants, myopathy, RNA sequencing, RYR1, whole‐genome sequencing

## Abstract

**Background:**

Defects in the *RYR1 (*OMIM#180901) gene lead to Ryanodine receptor type 1‐related myopathies (RYR1‐RM); the most common subgroup of congenital myopathies.

**Methods:**

Congenital myopathy presents a diagnostic challenge due to the need for multiple testing modalities to identify the many different genetic etiologies. In this case, the patient remained undiagnosed after whole‐exome sequencing (WES), chromosomal microarray, methylation analysis, targeted deletion and duplication studies, and targeted repeat expansion studies. Clinical whole‐genome sequencing (WGS) was then pursued as part of a research study to identify a diagnosis.

**Results:**

WGS identified compound heterozygous *RYR1* intronic variants, RNA sequencing confirmed both variants to be pathogenic causing RYR1‐RM in a phenotype of severe congenital hypotonia with respiratory failure from birth, neonatal brain hemorrhage, and congenital heart disease involving transposition of the great arteries.

**Conclusion:**

While there is an ongoing debate about the clinical superiority of WGS versus WES for patients with a suspected genetic condition, this scenario highlights a weakness of WES as well as the added cost and delay in diagnosis timing with having WGS follow WES or even ending further genetic testing with a negative WES. While knowledge gaps still exist for many intronic variants, transcriptome analysis provides a way of validating the resulting dysfunction caused by these variants and thus allowing for appropriate pathogenicity classification. This is the second published case report of a patient with pathogenic intronic variants in RYR1‐RM, with clinical RNA testing confirming variant pathogenicity and therefore the diagnosis suggesting that for some patients careful analysis of a patient's genome and transcriptome are required for a complete genetic evaluation. The diagnostic odyssey experienced by this patient highlights the importance of early, rapid WGS.

## 1 INTRODUCTION

The Ryanodine receptor type 1 (RYR1) gene (OMIM#180901) codes for the ryanodine calcium channel of the sarcoplasmic reticulum, which is involved in the excitation–contraction coupling of skeletal muscles. Upon membrane depolarization, conformational changes trigger calcium release through the ryanodine receptor and subsequent muscle contraction ensues (Neto et al., 2017). Pathogenic variants in this gene lead to Ryanodine receptor type 1‐related myopathies (RYR1‐RM); the most common subgroup of congenital myopathies (Jungbluth et al., 2016). The phenotypic spectrum of disease is broad and ranges from minimally progressive proximal muscle weakness and hypotonia at the milder end of the spectrum to joint contractures, scoliosis, ophthalmoplegia, and respiratory compromise in more severe cases, and a lethal form of fetal akinesia with deformation sequence at the extreme end of the spectrum (Alkhunaizi et al., 2019). Variable clinical and histopathological presentations have been described in both dominant and recessive inheritance patterns of the disease (Snoeck et al., 2015). Historically, RYR1‐RM was thought to have specific histological phenotypes based on these modes of inheritance. The autosomal dominant presentation was more typically associated with a milder phenotype, sometimes associated with malignant hyperthermia, and with histopathologic evidence of central core disease (Jungbluth, 2011). The recessive presentation was noted to have a more severe childhood‐onset of disease and was associated with multiminicore disease, centronuclear myopathy, or congenital fiber‐type disproportion (Amburgey et al., 2013). Continuing investigations into the genotypic and phenotypic spectrum of RYR1‐RM suggest that clear histopathological delineation may no longer be a reliable differentiation technique for diagnosis, as there appears to be histopathological overlap between autosomal dominant and autosomal recessive disease (Todd et al., 2018), though the autosomal recessive form of the disease does consistently show a more severe myopathic phenotype (Zhou et al., 2007).

## 2 CASE PRESENTATION

**FIGURE 1:**
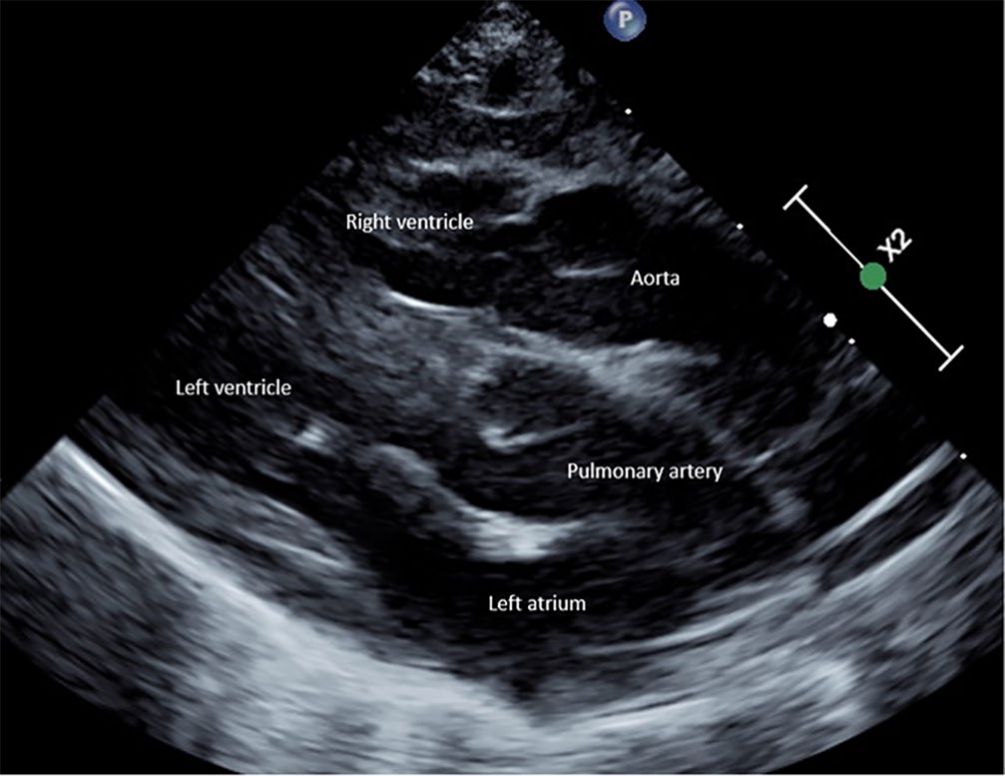
Parasternal long‐axis view on transthoracic echocardiogram in our patient demonstrating D‐transposition of the great arteries. The aorta is anterior and rightward relative to the pulmonary artery and arises from the right ventricle

The patient was a 38‐week gestation male born via spontaneous vaginal delivery to a healthy 24yo G2P2002 female following an uncomplicated naturally conceived pregnancy by non‐consanguineous union. Prenatal care was timely and appropriate, and testing, including routine ultrasounds, was normal. His birth weight was 3080 g and his initial Apgar scores were 1, 2, and 3 at 1, 5, and 10 minutes, respectively. He was intubated due to persistent hypoxemia and poor respiratory effort despite appropriate resuscitation. Following intubation, he remained profoundly hypoxic, and urgent echocardiography demonstrated d‐Transposition of the Great Arteries (d‐TGA) with an intact ventricular septum and restrictive atrial septum (Figure 1). A continuous infusion of prostaglandin E1 was initiated to maintain patency of the ductus arteriosus and he was transferred to the Cardiac Intensive Care Unit (CICU). Family history was notable only for the maternal history of bitemporal craniosynostosis, hearing loss, low to normal intelligence quotient, and attention deficit and hyperactivity disorder. There was no other family history of congenital anomalies, cognitive impairment, or childhood health problems.

**FIGURE 2:**
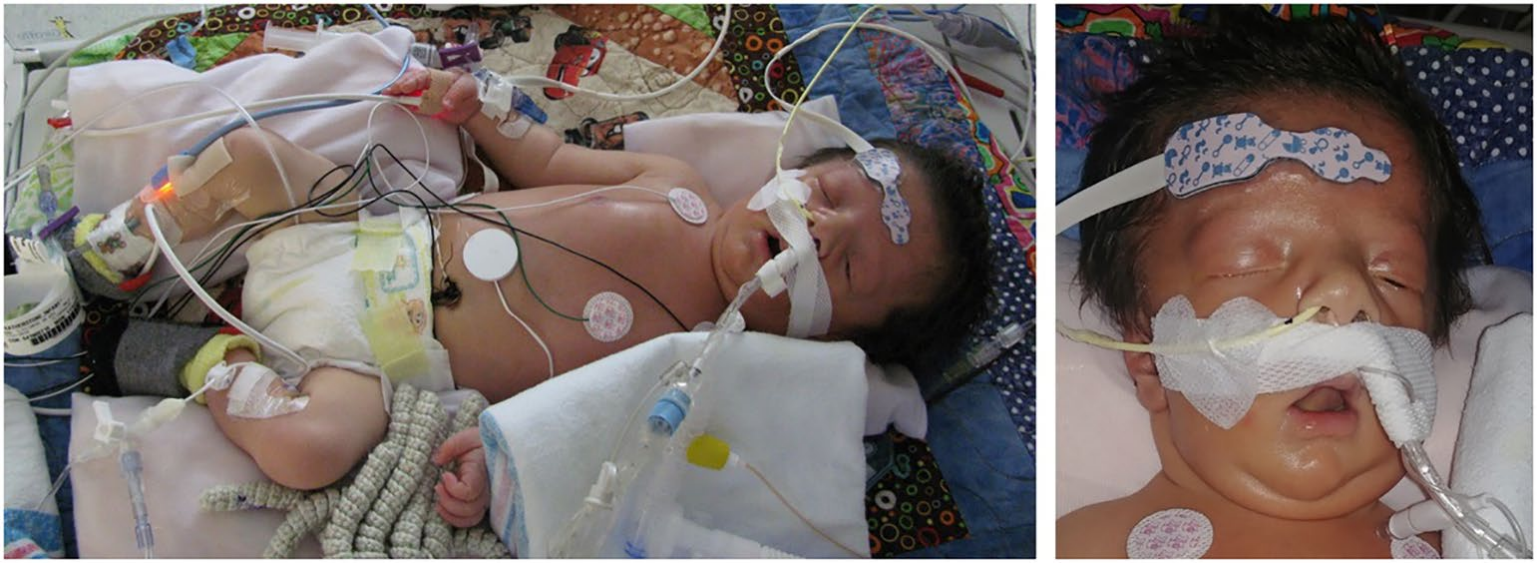
Frog‐leg position demonstrating hypotonia, facial features with downslanting eyes, high arched palate, downturned mouth corners, and open mouth low tone position

The patient's initial general exam showed bitemporal wasting, down‐slanting palpebral fissures, wide nasal bridge, tented upper lip, and high arched palate (Figure 2). His neurologic exam was significant for sluggish but symmetric pupillary reflex and a very weak suck. Posture was abnormal with frog‐leg position. There were no spontaneous movements. He was poorly responsive to painful stimuli with only minimal movement. He had a very weak reflexive palmar and plantar grasp. His tone was severely diminished axially and in all extremities. His deep tendon reflexes were absent and his Babinski reflex was mute.

**FIGURE 3:**
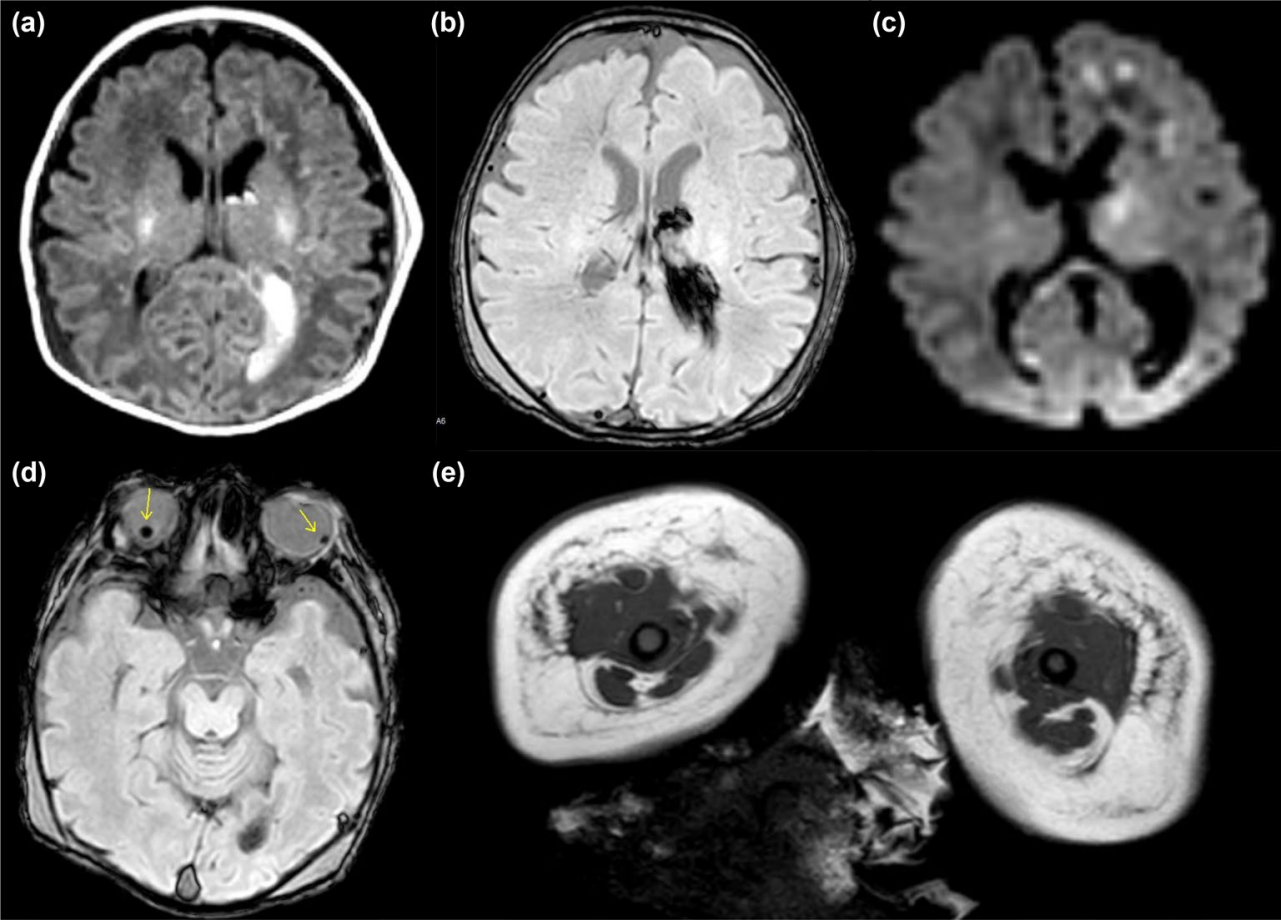
MRI Findings: T1, SWI, and DWI with hemorrhagic infarction (a–c). SWI with retinal hemorrhages (d) and thigh MRI with posterior atrophy (e)

A brain magnetic resonance imaging (MRI) obtained on day of life (DOL) 2 showed a left periventricular hemorrhagic venous infarction, left thalamic hemorrhage, distended left lateral ventricle, bilateral hemorrhagic white matter injury, and bilateral retinal hemorrhages (Figure 3). Four‐hour electroencephalography (EEG) was normal. The EEG was repeated on DOL 4 showing excessive intermittent sharp transients but no seizures. He also had normal creatine phosphokinase and thyroid‐stimulating hormone levels. His persistent poor tone and responsiveness were felt to be out of proportion to the MRI and EEG findings and his physical exam was suggestive of severe hypotonia starting in utero, prompting concerns for a congenital neuromuscular condition. He remained ventilator‐dependent throughout his course due to minimal respiratory effort.

Electromyography and nerve conduction studies demonstrated motor axonal peripheral polyneuropathy but were limited by electrical interference. A thigh MRI showed symmetric edema and atrophy of the abdominal wall and thigh musculature, predominantly seen in the posterior compartment (Figure 3). A quadriceps muscle biopsy showed marked myopathy with fiber size variation, and numerous immature type 2C muscle fibers. Electron microscopy showed abnormal sarcomeric organization and myofibril loss. No cores or rods were identified. Several esterase‐positive fibers were also noted suggesting a concomitant neurogenic component.

This patient had a heart defect that was incompatible with life without surgical intervention. He was initially palliated with a balloon atrial septostomy on DOL 0 and remained on prostaglandins throughout his clinical course. However, his neuromuscular disease and intraventricular and parenchymal hemorrhages prevented definitive surgical repair as it was felt he would not tolerate cardiopulmonary bypass. Thus, palliative care options were explored, and the patient was ultimately compassionately extubated and passed shortly thereafter. These decisions were based on the clinical presentation of severe neuromuscular disease before final genetic testing confirmed the diagnosis.

## 3 GENETIC TESTING

Chromosomal microarray was negative (DOL 28) for copy number variants or aneuploidy. *SMN1* sequencing and deletion/duplication analysis were negative (DOL 17). Methylation of chromosome 15q11.2 region was normal (DOL 24). Repeat expansions in *DPMK* were normal (DOL 24). Whole‐exome sequencing was nondiagnostic (DOL 24). Whole‐genome sequencing (Lab Rady Children's Institute, Edico DRAGEN pipeline, average genomic coverage 35X) on (DOL 28) identified a likely pathogenic variant in *RYR1* (Gene: *RYR1* Transcript: ENST00000359596 Genomic Coordinates: 19:38983277 Variant; c.6274+1G>A inherited maternally), along with the second variant of unknown significance in *RYR1* (Gene: *RYR1* Transcript: ENST00000359596 Genomic Coordinates: 19:39014507 Variant: c.10441‐48G>A inherited paternally), the variants were inherited in trans. A provisional diagnosis of RYR1‐RM was made in this case, given the phenotype and genotype findings. Further functional studies were pursued after the patient was deceased to confirm the diagnosis for the family.

Both variants identified in this case were intronic splice variants, and RNA sequencing became available as a clinical test after this patient was deceased. Preserved muscle samples from prior biopsy were sent for clinical RNA analysis (MNG Laboratories, Illumina Truseq Stranded Total RNA Library). Results returned confirming the pathogenicity of both variants. The c.6274+1G>A variant was detected in approximately 44% of the transcripts (7/16 reads) and caused a novel splice junction at chr19:38983254–38984992, predicted to lead to a change of the amino acid at position 2093 from serine to a valine and shift in the reading frame thereafter, with a stop codon predicted 40 codons beyond the change. The interpretation based on this functional testing continued to be likely pathogenic. The c.10441‐48G>A variant was detected in approximately 30% of the transcripts (10/33) and appeared to cause a novel splice junction at chr19:39013948–39014507 which was predicted to lead to a change of the amino acid at position 3841 from alanine to glycine and a shift in the reading frame thereafter, a termination stop codon is predicted three codons beyond this change. The available RNA functional data justified the classification of this variant as Likely Pathogenic.

## 4 HISTOLOGY

A biopsy of the quadriceps muscle was completed on DOL 36. Routine H&E stained sections showed skeletal muscle fibers with marked variation in fiber size, scattered fibers were mildly hypertrophic, the majority of fibers were quite variable in size. The Gomori trichrome stain showed no evidence of ragged red fibers or rimmed vacuoles, though there was a focally mild increase in endomysial connective tissue and scattered fibers containing irregular small magenta‐colored structures (though well‐formed rods were not appreciated). DPNH stain showed no well‐formed cores or tubular aggregates. ATPase at pH 10.0 and 4.6 both showed an intact checkerboard pattern of type 1 and 2 muscle fibers with no numeric fiber‐type disparity though there was a tendency for type 2 smallness. ATPase at pH 4.3 indicated the presence of immature (Type C) fibers. PAS and Oil‐Red‐O show normal quantity and distribution of glycogen and lipid respectively. Succinic acid dehydrogenase and cytochrome oxidase both showed normal quantity and distribution of mitochondria. Stains for merosin, alpha dystroglycan, and collagen 6 showed strong diffuse membrane staining throughout the skeletal muscle biopsy. Overall, the routine muscle pathology concluded that the sample exhibited myopathy with marked fiber size variation, numerous immature type 2C muscle fibers, and marked ultrastructural abnormalities. Further muscle pathology studies, such as western blot, were not feasible due to the small tissue sample with profoundly decreased muscle fibers.

**FIGURE 4:**
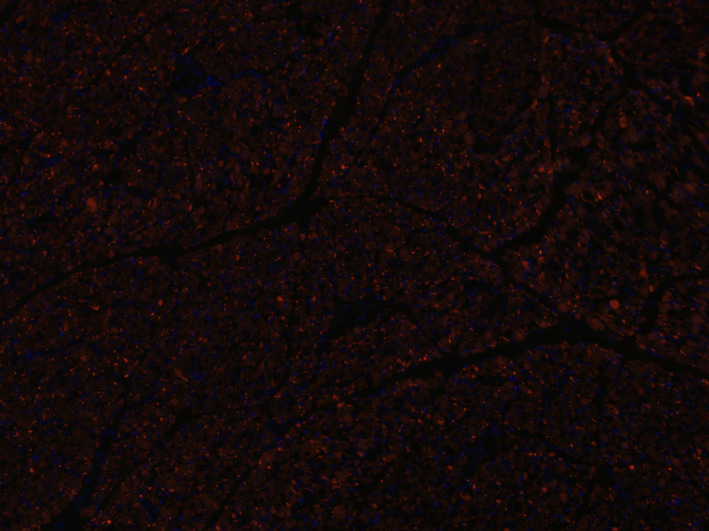
Patient muscle biopsy sample. 10× magnification with RYR1 ab IHC stain in Texas Red and DAPI for nuclei in blue

**FIGURE 5:**
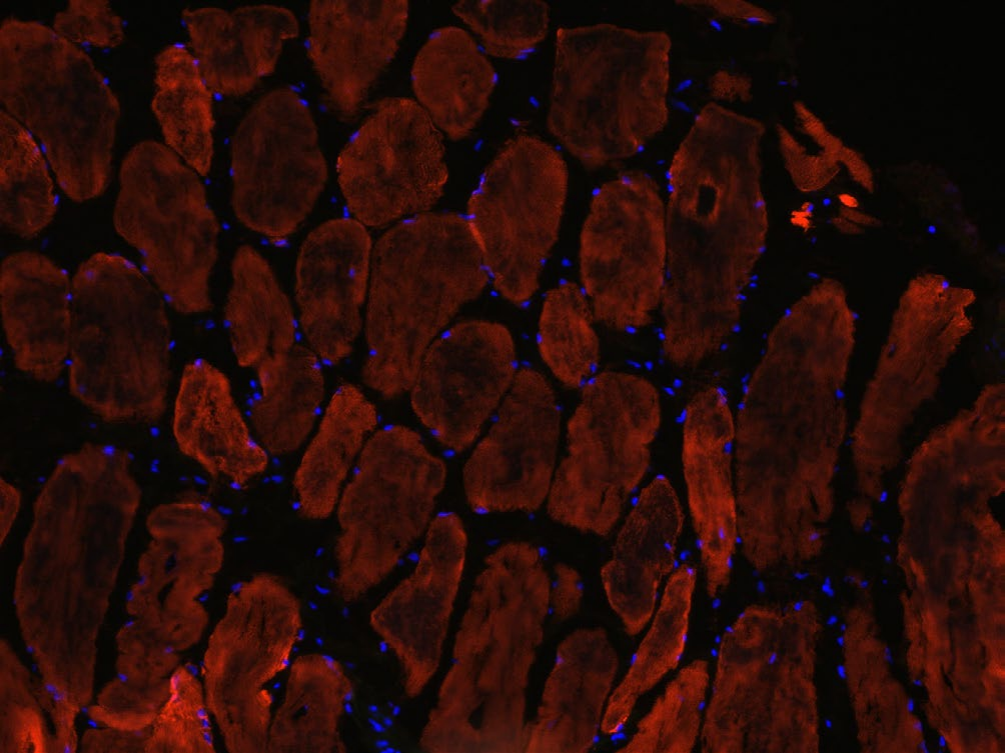
Control normal muscle biopsy sample. 10× magnification with RYR1 ab IHC stain in Texas Red and DAPI for nuclei in blue

Due to the preliminary pathology findings of myopathy, RYR1 (ab2868) antibody immunohistochemical staining was pursued to investigate the abnormalities in RYR1 density, location, and structure in the patient's muscle biopsy. The staining pattern for RYR1 in the patient's sample (Figure 4) was much reduced or absent altogether as compared to a control sample (Figure 5).

## 5 DISCUSSION

Neonatal hypotonia often presents a unique diagnostic challenge. The differential is very broad and includes myopathy, neuropathy, and neuromuscular junction disease, as well as central nervous system abnormalities. Neonatal hypotonia can be caused by copy number variants (charcot‐marie‐tooth), short tandem repeat triplet expansion disorders (myotonic dystrophy), abnormal DNA methylation patterns (Prader–Willi syndrome), whole‐exon deletions (spinal muscular atrophy), aneuploidy (Down syndrome), and single nucleotide variation (many myopathies, including nemaline, myotubular, and hyaline). Thus, one broad genetic test is often insufficient to interrogate all of the candidate genetic diagnoses. In many cases, an iterative approach starting with the highest yield test is reasonable. However, in this case, time was of the essence with a critical unrepaired heart lesion, thus testing for all options was performed in parallel to expedite a molecular diagnosis.

What constitutes an adequate genetic evaluation? When can we say there is not a genetic cause for a patient's symptoms, or at least not one that is diagnosable with the best diagnostic modalities at this time? Both questions have become more challenging to answer with the rapid evolution of genetic testing which now includes clinical analysis of an entire genome and various metabolomics, all on the backdrop of hundreds of new genetic disorders described every year (Boycott et al., 2017). This case highlights the challenges of a phenotype that leads to a broad differential diagnosis and requires multiple testing modalities to work through. Our approach to the diagnostic evaluation prior to the genome analysis can certainly be called thorough if not exhaustive. But, recent evidence has shown the superiority of genome to exome in variant detection but not necessarily a higher diagnostic yield (McCarthy, 2019). However, recent advances in WGS, including lower costs of sequencing and higher throughput in analysis, have made this test more accessible to clinicians evaluating rare genetic diseases. Indeed, WGS as a first‐line test, may overcome many of the superfluous and iterative test algorithms for complicated presentations such as congenital myopathy, in that it has the advantage of simultaneously interrogating copy number variation, short tandem repeats, exon deletion, and intronic variants, with overall better coverage at intron/exon junctions. When used as a first‐line test, it has an increased yield over WES, upwards of 15% in some studies (Lionel et al., 2018). A typical WES yields about 20,000 variants, typically about 10 genes with homozygous variants, 40–50 genes with compound heterozygous variants, and 250–300 with single heterozygous variants possibly related to a phenotype (Lee et. al, 2014). A further complication in the discovery of variants of uncertain significance (VUS), is the challenge of reclassification. A VUS at the time of initial report may become upgraded to likely pathogenic or pathogenic as more is learned about the genotype/phenotype, as more patients are identified by increased access and utility to genomic testing, and as more functional studies are performed. Indeed, as many as 38% of patients with a VUS finding at the time of initial test report had a variant upgraded to likely pathogenic or pathogenic within 5 years of the initial test (Roselle et. al, 2019). Unfortunately, automatic variant reclassification is not done as a standard of practice by labs due to challenges in reimbursement (Plon & Rehm, 2018), and typically requires initiation by a clinical team. Without proactive variant reanalysis efforts, the uncertainty of a genetic diagnosis remains for many patients.

One could imagine how these challenges are amplified as whole‐genome sequencing no longer focuses solely on exonic variants (~1% of the genome) as its predecessor the whole‐exome sequence did rather intronic variants (~24% of the genome) become the majority of SNVs identified, and much less is known about the intronic portion of the genome. In WGS, the entire genome is evaluated; the sequenced DNA jumps from 2% to 98% of the genome, with more than 3 million SNVs per WGS sample to interpret (Anath et al, 2018). This poses a new challenge for interpretation of these variants; though a robust database of exonic variants is constantly evolving, information on intronic variants is lacking (Smedley et. al, 2016).

One method for better understanding intronic variants, particularly those involving a splice site, is through RNA analysis. Clinical RNA sequencing is in its infancy, but evidence suggests that as many as 30% of disease‐causing variants impact RNA expression and/or processing and are found in non‐coding regions (Gonorazky et al., 2019). RNA sequencing provides transcript‐level information for interpreting splice changes. By sequencing the RNA, we are able to capture post‐transcriptional real‐time functional data on how splicing alterations impact coding sequences, and thus are able to make more confident interpretations on the pathogenicity of intronic sequence variants. In this case, what would easily be considered the current standard of care and by all considerations, a robust workup, would not have identified a diagnosis for this patient. However, cutting‐edge WGS and RNA sequencing were able to confirm a diagnosis. Both technologies are rapidly establishing their clinical utility and this case is a perfect example of why WGS is becoming the first test to send in a critically ill neonate with a complex phenotype.

Once a genetic diagnosis was confirmed, additional questions required consideration. While myopathy leading to congenital hypotonia and respiratory insufficiency is a well‐known phenotype of the autosomal recessive form of RYR1‐RM, it is less clear how other aspects of the patient's phenotype relate to the genotype. Are CNS hemorrhages part of the phenotype? Is congenital heart disease part of the RYR1‐RM phenotype or was this unrelated to the genotype and second to another possibly multifactorial etiology? Indeed, prenatal and neonatal hemorrhages are a rare finding in RYRI‐RM as was seen in a newborn with autosomal recessive RYR1‐RM due to compound heterozygous mutations (Brackmann et al., 2018). Mild bleeding abnormalities have been reported in the dominant form of RYR1‐RM in patients experiencing malignant hyperthermia (Lopez et. al, 2016) as well as by a knock‐in mouse model, MHS RYR1Y522 (Lopez et. al, 2016) There is evidence that this bleeding is caused by smooth muscle dysfunction in the vasculature and may be reversed with dantrolene (Lopez et. al, 2016). While clues to the bleeding connection were evident, no previous case reports of congenital heart disease and RYR1‐RM existed in our literature review. The RYR1 receptor is known to play an important role in excitation–contraction coupling in striated muscle but has not been reported to have that same role in cardiac muscle (Amador et al., 2013). A cardiac isoform of the ryanodine receptor does exist, RYR2, and mutations in this gene lead to a phenotype of catecholaminergic polymorphic ventricular tachycardia (CPVT) and a cardiomyopathy syndrome known as arrhythmogenic right ventricular dysplasia type 2 (ARVD2) (Amador et al., 2013). The phenotype for each ryanodine receptor disease seems to be unique to the receptor and tissue type; cardiomyopathy is not reported in the phenotypic spectrum of RYR1‐RM; and skeletal myopathy is not reported in the phenotype of RYR2 disease (Olubando et al., 2020). Even if there was crossover in the function of RYR1 to cardiac tissue, the phenotype one might expect would be cardiomyopathy. Given this historical information, structural congenital heart disease would not be attributed to pathogenic *RYR1* variants.

Interestingly, Zvaritch et al. (2007) generated a knock‐in mouse line carrying the murine analogous *RYR1* homozygous I4895T mutation. These mice, with an autosomal recessive RYR1‐RM disease presentation, showed a phenotype of reduced muscle mass, and indeed were paralyzed from birth, eventually dying of respiratory failure and asphyxia. Interestingly, these homozygous mice exhibited abnormal cardiovascular development. Neonatal mice demonstrated a delay in cardiac development; namely their hearts retained the early embryonic antero‐posterior orientation instead of the oblique orientation that is characteristic of later‐stage fetuses. This abnormal cardiac positioning precluded the proper formation of the outflow tract, interfering with bending of the arterial arch and pulmonary trunk, leading to congenital heart defects of the great vessels. Failed cardiac rotation in the human embryo equivalent would present with conotruncal defects, including transposition of the great vessels (Angelini, 1995).

Additional evidence supporting the possibility of congenital heart disease being part of the RYR1 phenotypic spectrum of disease comes from a large study evaluating 2,871 patients with congenital heart disease using WES. Recessive genotypes were enriched in their cohort, including genes involved in muscle cell development, *KEL*, *MYH6*, *MYH11*, *NOTCH1*, *and RYR1* (Jin et al., 2017). Among patients in this cohort, three had compound heterozygous mutations in *RYR1* (Y246C/P3429H; R1231H/R3672S; P429L/A882V, and their cardiac phenotypes were TGA in the former and LVOTO (left ventricular outflow tract obstruction), in the latter two patients (Jin et al., 2017). Other clinical information, including myopathy phenotype, were not described in this study, though prediction software suggests many of these *RYR1* variants are likely pathogenic, or VUS with damaging predictions (Kopanos et al., 2019). Though this evidence seems to suggest a possible link between *RYR1* variants and conotruncal heart defects, further evidence supporting the link runs short. In a large study of 66 family trios with a phenotype of d‐TGA, none were found to have *RYR1* variants on whole‐exome sequencing (Liu et al., 2020). This study had limited sensitivity for intronic variants though, a possible limitation.

Thus, given our patient with congenital heart disease involving d‐TGA with confirmed compound heterozygous pathogenic variants in RYR1 and no other putative variants for conotruncal heart defects identified on either WES or WGS for our patient, large congenital heart disease cohorts with patients identified having coexisting conotruncal heart defects and compound heterozygous mutations in *RYR1*, and knockout murine mouse models with conotruncal heart defects, we propose that congenital heart disease may be a phenotypic feature attributable to RYR1‐RM, though more evidence supporting or refuting this assertion is warranted.

In summary, this patient eloquently highlights the challenges of making a genetic diagnosis both when the phenotype is non‐specific and also when the disease gene itself has a wide‐ranging phenotypic spectrum, multiple modes of inheritance, and novel variants that have yet to be adequately characterized. What constitutes an adequate genetic evaluation remains a moving target, but mounting evidence (Petrikin et. al, 2018) suggest WGS is not only the penultimate test but should be the first test sent in a critically ill neonate with a suspected genetic condition and a non‐specific phenotype. In our patient, it was the last test sent in what was a relatively short diagnostic odyssey compared to most but was the only test that could have made the diagnosis. With the help of RNA sequencing, we were able to upgrade what was a VUS to a likely pathogenic variant and expand the genotype of RYR1‐RM.

As with any genetic diagnosis, these findings provided much‐needed answers to a family that very much wanted to understand the cause of their child's condition, and also provided important information about future family planning. Ultimately, functional testing allowed us to confirm a diagnosis of RYR1‐RM for this family nearly 18 months after their child had passed. The family nearly cried with relief at these results, for 18 months this patient's mother had struggled with occasional lingering feelings of guilt in having to make the decision to redirect care based on the clinical picture and a provisional (but not molecularly confirmed) diagnosis. She had always wondered if an alternative diagnosis, with a therapy, may have existed, and that she didn't give the clinical team enough time to find this diagnosis. Having a molecular diagnosis offered this family peace of mind, finally.

## CONFLICT OF INTEREST

None of the listed authors have any reported conflicts of interest, and nothing to disclose.

## AUTHORS’ CONTRIBUTION

AS prepared the majority of the manuscript including the background, genetic testing, and discussion. AZ prepared the majority of the case report and physical exam findings. TP provided cardiac case description and imaging. RH, CT, and DC provided mentorship and review during the writing process. KS organized the overall conceptualization of paper and provided ongoing editing and revisions.

## ETHICS APPROVAL AND CONSENT TO PARTICIPATE

Written consent to participate and written consent for photographs was received from the subject of this case report.

## CONSENT FOR PUBLICATION

Written informed consent was obtained from the patient's parent for publication of this Case Report and any accompanying images and videos.

## Data Availability

Not applicable. All data generated or analyzed during this study are included in this published article. The data that support the findings of this study are available from the corresponding author upon reasonable request.
